# Niacin (Vitamin B3)-Induced Acute Fulminant Hepatic Failure in a 24-Year-Old Female

**DOI:** 10.7759/cureus.69518

**Published:** 2024-09-16

**Authors:** Michael Duffy, Gary Zhang, Chanika Ariyawansa, Matthew H Anstey

**Affiliations:** 1 Intensive Care Medicine, Sir Charles Gairdner Hospital, Perth, AUS; 2 Laboratory Medicine, Sir Charles Gairdner Hospital, Perth, AUS; 3 Intensive Care Medicine, The University of Western Australia, Perth, AUS

**Keywords:** acute fulminant liver failure, hepatology, intensive care unit (icu), niacin, toxicology and poisoning

## Abstract

Acute liver failure (ALF) is a rare, life-threatening condition characterized by acute severe liver injury, encephalopathy, and coagulopathy in the absence of prior liver disease. The causes of ALF are broad and varied worldwide, commonly including triggers such as drugs (predominantly paracetamol) in developed countries and viral infections in developing nations. Prompt diagnosis and management are crucial in acute fulminant liver failure as highlighted in this case of a 24-year-old female with ALF secondary to vitamin B3 overdosing.

This study further highlights the need for a high degree of clinical suspicion that physicians need to ascertain the cause of acute liver failure, the complexity of its management, and the significant harm unnecessary dietary supplementation can result in. This is a crucial example of why healthcare professionals need to educate their patients about the potential adverse consequences of dietary supplements.

## Introduction

Dietary supplementation has become increasingly common over the past few decades, with some reports from the United States claiming that 70% of people take dietary supplements daily [1,2]. Despite these high rates, it has been reported that only 23% of these supplements have been recommended by healthcare professionals [3,4]. The perception that over-the-counter supplements are harmless may contribute to their widespread use.

Niacin (nicotinic acid) is a naturally occurring water-soluble organic compound found in meat, poultry, and redfish. It is one of the several forms that vitamin B3 takes to perform its functions. The Food and Nutrition Board of America recommends a daily intake of 14 mg of niacin for a non-pregnant female greater than the age of 18 years. It functions as a co-enzyme, aiding over 400 different reactions in the human body for a wide range of processes, such as the organic synthesis of cholesterol and the repair of DNA [5,6]. High-dose niacin (>500 mg) as a monotherapy or in combination with statins has been used for the treatment of hypertriglyceridemia and hypercholesterolemia with little evidence in the reduction of all-cause mortality and cardiovascular events [7]. There are examples of case reports and naturopathic providers suggesting the benefit of niacin supplementation for mood disorders [8]. There is also some low-level evidence of an association between niacin levels and depression in adults which may encourage patients to try supplementing it to ameliorate low moods [9].

There have been no reported side effects of niacin when taken at dietary levels. Flushing is the most common side effect of high dosages (50 mg/day) of niacin but is generally transient. When taken in higher dosages (>1,000 mg daily), side effects such as hypotension, fatigue, GI dysmotility, and hepatotoxic injury may be observed when taken over a prolonged period [6]. Niacin is metabolized in the liver by both amidation and conjugation pathways. The conjugation pathway has a high capacity and results in the formation of prostaglandins, which causes flushing. The amidation pathway is a low-capacity, multistep reaction resulting in the formation of nicotinamide adenine dinucleotide, which disrupts mitochondria and causes hepatotoxicity through hepatic cell apoptosis [10].

We present a case of a 24-year-old female who developed acute liver failure after taking high doses of niacin (>1,000 mg/day) for several months to manage her anxiety and depression on the recommendation of both her psychiatrist and naturopath. This study highlights the potential risks associated with nutritional supplement ingestion.

## Case presentation

A 24-year-old Caucasian female with a four-year history of generalized anxiety disorder, depression, and migraines presented to the emergency department with generalized epigastric pain, nausea, and fatigue. She had presented two days prior with the same symptoms but was discharged with a reassuring physical examination and unremarkable venous blood gas.

The patient was initially prescribed a vitamin B complex by a holistic psychiatrist but, in the preceding few months, had increased her intake of vitamin B3 on the advice of a naturopath to alleviate her anxiety and low mood. She had been taking approximately two or three 500 mg tablets per day of niacin for roughly two months before the presentation. To control panic attacks, she had taken 5 g of niacin on several days in the week before her hospital admission. She did not have a history of liver disease, alcohol consumption, paracetamol use, illicit drug use, or other acute illnesses before the onset of the symptoms. She was not taking any medication that would interact with niacin or which had any potential hepatotoxic effect. She had no suicidal ideation or previous history of attempted suicide.

The only remarkable elements of her initial physical assessment were generalized abdominal pain and sinus tachycardia. Blood tests showed significantly deranged liver function tests and coagulation studies (Table 1). Both the renal function tests and inflammatory markers were moderately deranged (Table 1).

**Table 1:** Summary of initial blood tests of the patient on arrival to the emergency department with reference ranges included in brackets.

Full blood count	Liver function tests	Coagulation studies	Renal function and CRP	Electrolytes	Arterial blood gas	Other
Hemoglobin 156 g/L (115-160 g/L)	Bilirubin 134 µmol/L (<20)	Prothrombin M2 >180 s (12-16.5 s)	Urea 2.6 mmol/L (3-8 mmol/L)	Potassium 4.2 mmol/L (3.5-5.2 mmol/L)	pH 7.44 (7.35-7.45)	Lipase 540 U/L (<60 U/L)
White cell count 16.9 x 10^9^/L (4-11 x 10^9^/L)	Albumin 39 g/L (35-50 g/L)	INR >10 (0.9-1.3)	Creatinine 101 mmol/L 45-90 mmol/L)	Sodium 138 mmol/L (135-145 mmol/L)	pCO_2_ 38 mmHg (36-35 mmHg)	Hepatitis and HIV screens negative
Neutrophils 14.82 x 10^9^/L (2-7.50 x 10^9^/L)	Alanine transaminase 1,43,000 U/L (<35 U/L)	APTT 73.2 s (27.5-38.5 s)	GFR 67 mL/min/1.73 m^2^ (>90 mL/min/1.73 m^2^	Adjusted calcium 2.49 mmol/L (2.10-2.6 mmol/L)	pO_2_ 82 mmHg (85 -110 mmHg)	B-HCG negative
Lymphocytes 0.47 x 10^9^/L (1.20-4 x 10^9^/L)	Alkaline phosphatase 218 U/L (30-110 U/L)	Fibrinogen 1.1 g/L (2-4 g/L)	CRP <1 (<5 mg/L)	Magnesium 0.65 mmol/L (0.70-1.10 mmol/L)	Bicarbonate 25 mEq/L (21-28 mEq/L)	Lactate dehydrogenase 1,111 U/L (90‐220 U/L)
Monocytes 1.65 x 10^9^/L (0.2-1 x 10^9^/L)	Gamma-glutamyl transferase 75 U/L (<40 U/L)	-	Lactate 5.6 mmol/L (<2 mmol/L)	Phosphate 0.40 mmol/L (0.75-1.50 mmol/L)	Lactate 6.5 mmol/L (2 mmol/L)	Creatinine kinase 52 U/L (25-145 U/L)

Both the ultrasound abdomen pelvis and contrast-enhanced CT abdomen pelvis showed normal hepatic vascular supply and no ductal dilatation. Paracetamol levels were <10 µg/mL; hepatitis A, B, and C serologies were negative; and ammonia level was 49 µmol/L (reference interval {RI}: 11‐48 µmol/L). A transthoracic echocardiogram demonstrated an ejection fraction of 65% with normal left ventricular function and no valvular pathology.

Several hours after she was initially admitted to the emergency department, the patient began to become hemodynamically unstable, with a systolic blood pressure of 70 and a blood glucose level of 3 mmol/L (RI 3-7.8 mmol/L for random glucose level). At the time, she was disorientated with some mild confusion, and her ammonia level was 122 µmol/L (<50 µmol/L). After discussion with acute toxicology services, the working diagnosis was fulminant acute liver failure with multiorgan dysfunction secondary to staggered supratherapeutic niacin ingestion. Investigations for other causes of liver failure (hepatitis screen, ceruloplasmin level, autoimmune screen) were negative. Interestingly, liver function tests taken with her general practitioner one month earlier were unremarkable. She was started on a N-acetyl cysteine (NAC) infusion, and due to the need for inotropic support, the patient was transferred to the intensive care unit (ICU).

During her admission to the ICU, she was started on broad-spectrum antibiotics (meropenem and anidulafungin) for empiric coverage of ongoing fevers and ammonia reduction therapy for the treatment of the immune dysfunction associated with acute liver failure (ALF). Her liver function tests were monitored six hourly. Multidisciplinary discussion between toxicology, ICU, and hepatology was ongoing. The patient was pre-emptively started on continuous veno-venous hemodiafiltration (CVVHDF) for liver failure and the potential for vitamin B3 removal. Table 2 outlines the change in serum niacin levels after CVVHDF was started. Two liters of dialysis effluent were sent for analysis on the first day of dialysis (second day of admission), and levels of 0.13 mg of nicotinamide were found (Table 2). Urine collected over 14 hours at the same time had 12.69 mg of nicotinamide. While testing for nicotinamide was not routinely available at this laboratory, analysis was performed using a preliminary mass spectrometry method on a Waters ACQUITY QDa Mass Detector (Milford, MA: Waters Corp.). She received a total of six days of CVVHDF with a dose of 70 mL/kg of effluent. Her significant coagulopathy was treated with 31 units of cryoprecipitate, 12 units of fresh frozen plasma, three units of platelets, and one liter of 20% albumin over four days. The patient was assessed for and placed on the liver transplantation list. The low niacin levels in the serum can be attributed to the short half-life of niacin (approximately 45 minutes) and the fact that the patient had not consumed food or the niacin tablets for approximately three days prior to her presentation due to abdominal pain and significant vomiting. This sample sent on the second day was also post-hemodialysis.

**Table 2:** Patient’s liver function tests, INR, and nicotinamide levels from day one of her admission including post-discharge results. ALT: alanine transaminase; GGT: gamma-glutamyl transferase; AST: aspartate aminotransferase; ALP: alkaline phosphatase; INR: international normalized ratio

Admission day	Total bilirubin (<20 µmol/L)	ALT (7-56 U/L)	Ammonia (11-32 µmol/L)	GGT (5 40 U/L)	AST (8-33 U/L)	ALP (44-147 U/L)	INR (0.8-1.1)	Serum nicotinamide levels (mg/L) (0.50-8.45 mg/L)
1	134 µmol/L	14,300 U/L	122 µmol/L	75 U/L	10,900 U/L	218 U/L	>10	-
2	113 µmol/L	9,620 U/L	120 µmol/L	51 U/L	6,270 U/L	164 U/L	2.5	0.03 mg/L
3	182 µmol/L	8,510 U/L	109 µmol/L	45 U/L	1,640 U/L	157 U/L	8.2	0.004 mg/L
4	285 µmol/L	4,500 U/L	87 µmol/L	34 U/L	None	151 U/L	6.5	0.002 mg/L
5	372 µmol/L	3,100 U/L	80 µmol/L	27 U/L	162 U/L	150 U/L	3.0	0.002 mg/L
6	290 µmol/L	1,610 U/L	65 µmol/L	26 U/L	55 U/L	122 U/L	3.7	0.001 mg/L
7	247 µmol/L	673 U/L	43 µmol/L	31 U/L	55 U/L	108 U/L	2.3	0.001 mg/L
19 (discharge)	291 µmol/L	199 U/L	27 µmol/L	68 U/L	138 U/L	122 U/L	1.2	-
30	349 µmol/L	115 U/L	25 µmol/L	70 U/L	139 U/L	128 U/L	1.1	-
55	69 µmol/L	103 U/L	23 µmol/L	123 U/L	103 U/L	129 U/L	1.1	-

**Figure 1:**
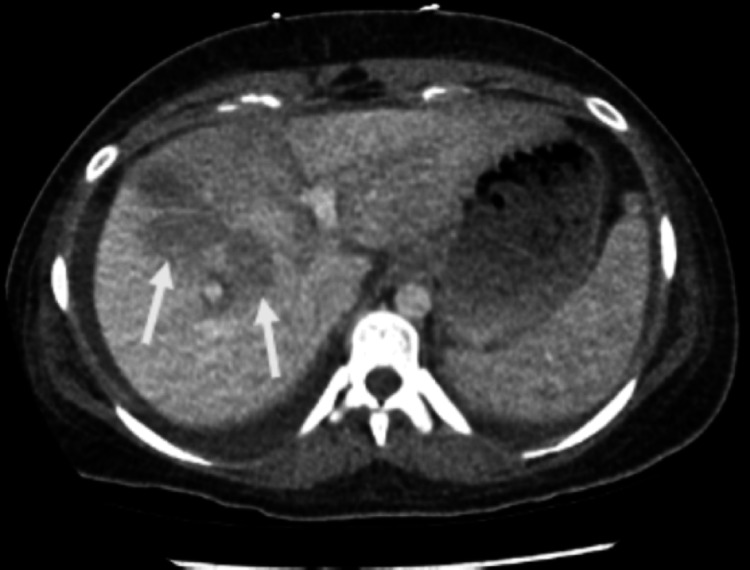
Axial view of a triple-phase CT abdomen with evidence of patchy necrosis indicated by arrows.

**Figure 2:**
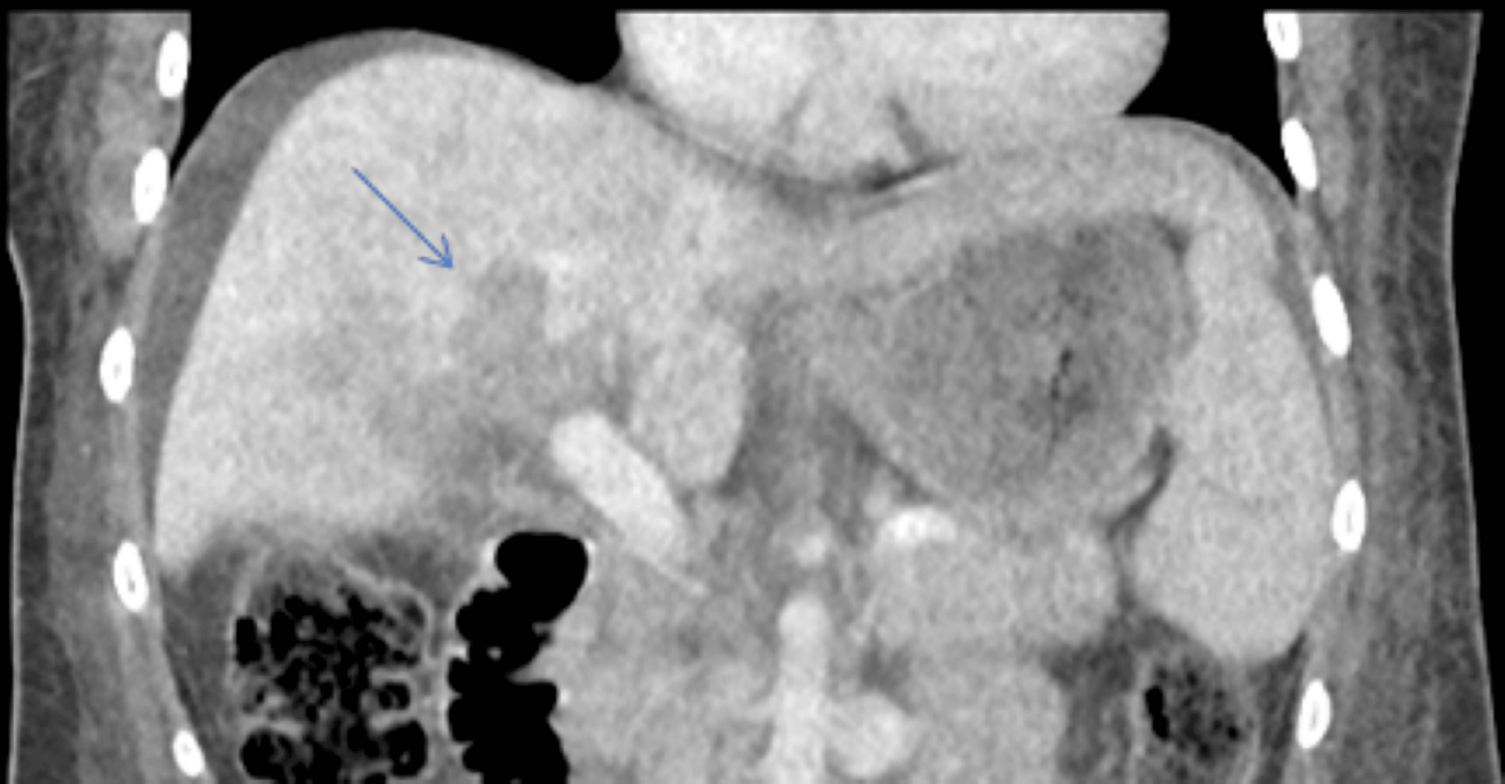
Coronal view of a triphasic CT abdomen pelvis with areas of patchy necrosis indicated by arrow.

A repeat triple-phase CT abdomen pelvis four days into her admission revealed areas of patchy perfusion throughout her liver visible in Figures 1, 2. Furthermore, a liver biopsy revealed subacute necrosis with a loss of 90% of hepatocytes in the sampled tissue. After a one-week ICU admission, the patient was transferred to a hepatology ward due to hemodynamic stability, improved liver function, and coagulation tests. Prior to discharge, she had ongoing input from hepatology, psychiatry, and dietetics with close outpatient follow-up. Her liver function testing was completely resolved after subsequent outpatient monitoring.

## Discussion

Acute fulminant liver failure (ALF) can be defined as any patient presenting with hepatic-induced coagulopathy and encephalopathy in individuals with no previous history of cirrhosis/liver disease [11]. There are approximately 2,000 cases of acute liver failure in the United States per year, and approximately 60% of these cases are caused by paracetamol. The worldwide incidence of ALF varies greatly depending on factors such as economic development and access to medication, but it is estimated to be approximately one case per million. Despite its low relative incidence rates, ALF requires significant resources and results in a high burden of disease on medical systems [12,13]. The relative rarity of ALF and the fact that potential precipitants can range from drug-induced and viral to metabolic disorders can make ascertaining the exact cause a challenge for any clinician. The broad range of differentials also adds to the potential difficulty associated with its treatment. The non-specific symptoms, such as nausea and vomiting, that are present early in the disease process may result in delayed presentations, further complicating treatment.

The rarity of ALF secondary to niacin means that there needs to be more evidence-based treatment guidelines that clinicians can consult. Dialysis was provided for this patient to help treat the liver failure and to see whether it could enhance the clearance of niacin. Despite the water solubility of vitamin B3, Wang et al. found no conclusive evidence that vitamins B1, 3, 5, and 6 were effectively dialyzed with high flux dialysis [14]. Our laboratory analysis of the dialysis effluent also suggests that it is not significantly cleared by dialysis. Nonetheless, the high-dose dialysis provided can be beneficial for all cases of acute liver failure likely due to its reduction in ammonia levels [15].

The exact cause of coagulopathy in ALF is multifactorial owing to impaired synthesis of procoagulant factors, disruption of fibrinolytic systems, deranged number/function of platelets, and occasionally a component of disseminated intravascular coagulation [16]. Our patient showed evidence of significantly deranged coagulation and liver studies even when compared to other similar case reports [17,18]. Staggering doses of drugs over days rather than a large dose at a single time can increase the risk of death in drug-induced liver injury and likely has a more significant overall impact on liver injury [19].

The high rates of vitamin supplementation in the community, while usually harmless, combine to create a situation where patients are harmed by non-beneficial supplements. Some reports indicate that 63% of adults in the United Kingdom have reported using the Internet for health-related information. Factual healthcare information online can benefit patients by informing them about their health and helping them access potential support networks. Potential negatives include increased anxiety through false information and the potential for health/financial damage through the sales of medications with little evidence to support them [20]. Cases like the one we presented highlight the need for physician/pharmacist-led education on dietary supplementation and increased regulations on health-related online information.

This represents a rare example of niacin-induced acute liver failure in the setting of mental health treatment. This study offers a broad range of learning opportunities, such as the potentially hazardous side effects of seemingly innocuous supplements, the wide range of differentials for acute liver failure, and the overall complexity in the management of drug-induced acute liver failure. The relatively short period (August-November) during which this presentation was developed highlights the need for greater education on the potential harms of unregulated dietary supplementation and the need for honest patient-doctor conversations about proper use.

## Conclusions

Niacin (vitamin B3 or nicotinic acid) is an essential human nutrient. It has been used in various settings, including traditional management of dyslipidemia, pellagra, and, more recently, in preventing non-melanoma skin cancers. However, it has also been used for migraines and anxiety, although the evidence base is poor. Niacin toxicity may manifest as flushing and itching in milder cases, and, in rare cases, as hypotension, hypoglycemia, and hepatotoxicity. No case reports of vitamin B3 overdose related to its use for anxiety have been published since 1980. This is an important example of the potential harm that the use of dietary supplements can cause when used inappropriately and should be used to emphasize the potential risks associated with unnecessary supplementation.
